# Vascular network expansion, integrity of blood–brain interfaces, and cerebrospinal fluid cytokine concentration during postnatal development in the normal and jaundiced rat

**DOI:** 10.1186/s12987-022-00332-0

**Published:** 2022-06-07

**Authors:** Sandrine Blondel, Nathalie Strazielle, Amel Amara, Rainui Guy, Christine Bain, Alix Rose, Laurent Guibaud, Claudio Tiribelli, Silvia Gazzin, Jean-François Ghersi-Egea

**Affiliations:** 1 BIP Facility, Lyon Neurosciences Research Center, Bron, France; 2 Brain-i, Lyon, France; 3 Fluid Team Lyon Neurosciences Research Center, INSERM U1028, CNRS UMR5292, Lyon University, Bron, France; 4 Active Biomarkers, Lyon, France; 5 Fondazione Italiana Fegato-Onlus, AREA Science Park, Basovizza, Trieste, Italy

**Keywords:** Blood–brain barrier, Blood–CSF barrier, Choroid plexus, Cerebrospinal fluid, Neurovascular, Postnatal development hyperbilirubinemia, Jaundice, Gunn, Cytokines

## Abstract

**Background:**

Severe neonatal jaundice resulting from elevated levels of unconjugated bilirubin in the blood induces dramatic neurological impairment. Central oxidative stress and an inflammatory response have been associated with the pathophysiological mechanism. Cells forming the blood–brain barrier and the choroidal blood–CSF barrier are the first CNS cells exposed to increased plasma levels of unconjugated bilirubin. These barriers are key regulators of brain homeostasis and require active oxidative metabolism to fulfill their protective functions. The choroid plexus-CSF system is involved in neuroinflammatory processes. In this paper, we address the impact of neonatal hyperbilirubinemia on some aspects of brain barriers. We describe physiological changes in the neurovascular network, blood–brain/CSF barriers integrities, and CSF cytokine levels during the postnatal period in normobilirubinemic animals, and analyze these parameters in parallel in Gunn rats that are deficient in bilirubin catabolism and develop postnatal hyperbilirubinemia.

**Methods:**

Gunn rats bearing a mutation in UGT1a genes were used. The neurovascular network was analyzed by immunofluorescence stereomicroscopy. The integrity of the barriers was evaluated by [^14^C]-sucrose permeability measurement. CSF cytokine levels were measured by multiplex immunoassay. The choroid plexus-CSF system response to an inflammatory challenge was assessed by enumerating CSF leukocytes.

**Results:**

In normobilirubinemic animals, the neurovascular network expands postnatally and displays stage-specific regional variations in its complexity. Network expansion is not affected by hyperbilirubinemia. Permeability of the blood–brain and blood–CSF barriers to sucrose decreases between one- and 9-day-old animals, and does not differ between normobilirubinemic and hyperbilirubinemic rats. Cytokine profiles differ between CSF and plasma in all 1-, 9-, and 18-day-old animals. The CSF cytokine profile in 1-day-old animals is markedly different from that established in older animals. Hyperbilirubinemia perturbs these cytokine profiles only to a very limited extent, and reduces CSF immune cell infiltration triggered by systemic exposure to a bacterial lipopeptide.

**Conclusion:**

The data highlight developmental specificities of the blood–brain barrier organization and of CSF cytokine content. They also indicate that a direct effect of bilirubin on the vascular system organization, brain barriers morphological integrity, and inflammatory response of the choroid plexus-CSF system is not involved in the alteration of brain functions induced by severe neonatal jaundice.

**Supplementary Information:**

The online version contains supplementary material available at 10.1186/s12987-022-00332-0.

## Introduction

The brain develops in a controlled environment secured by the blood–brain barrier located at the endothelium of the cerebral microvessels and by the blood–CSF barrier located at the epithelium of the choroid plexuses [1, 2]. The development of the vascular network is intimately linked to that of the neuronal network [3]. The choroid plexus-CSF system is a key element of neuroimmune regulation [4], and is involved in the neuroinflammatory response following perinatal injuries, in part through the secretion of multiple cytokines an chemokines [5]. Apart from these immune functions, these molecules fulfill important physiological functions in the context of brain development [6]. Severe neonatal jaundice results from liver immaturity that leads to increased levels of unconjugated bilirubin in the blood. This condition specifically affects the brain and can induce neurological impairment ranging from mild neurodevelopmental disorders to dramatic kernicterus [7–9]. The free (plasma protein unbound) fraction of unconjugated bilirubin circulating in the blood increases during severe hyperbilirubinemia. Free bilirubin, being lipophilic, easily penetrates into the central nervous system (CNS) by diffusion across brain barrier cells, and reaches intracerebral concentrations that lead to neurologic alterations. The neuropathological mechanisms associated with this condition remain elusive. Experimental evidence points to the occurrence of oxidative stress and inflammation directly or indirectly induced by unconjugated bilirubin in the brain, both events affecting neural cell proliferation, differentiation and apoptosis [10–15]. Cells forming the blood–brain interfaces require active oxidative metabolism to maintain their tightness and fulfill their functions of protection towards the maturing brain. These cells are the first CNS cells exposed to increased plasma levels of unconjugated bilirubin, and accordingly cerebral endothelial cells have been considered as targets for bilirubin toxicity (reviewed in [16]). In vitro, bilirubin was shown to lead to endothelial apoptosis and/or to the secretion of factors that are pro-angiogenic and induce barrier dysfunctions [16–19].

We therefore questioned whether pathologic neonatal hyperbilirubinemia alters the postnatal neurovascular network development, and blood–brain and blood–CSF barrier integrity, which would in turn impact brain maturation and functions. Because the choroid-plexus–CSF system is central to neuroimmune interactions and to the initiation of neuroinflammation, we further hypothesized that pathological hyperbilirubinemia would elevate CSF cytokine levels, sustaining neuroinflammation and impacting brain development. To test these hypotheses, we used the Gunn rat characterized by a deficiency in bilirubin conjugation induced by a spontaneous mutation in the 4th common exon of the UDP-glucuronosyltransferase UGT1a enzymes [20]. Homozygous pup rats, born from heterozygous mothers, display normal plasma levels of unconjugated bilirubin at birth, that sharply increase a few hours after birth and up to 17 days [21]. This model therefore recapitulates nicely the postnatal profile of unconjugated bilirubin concentration in plasma observed in babies with severe jaundice. The hyperbilirubinemic animals are characterized by a cerebellar hypoplasia and altered behavioral capacities [22].

In this paper we evaluated the complexity of the vascular network, the integrity of blood–brain interfaces using sucrose as a polar, non-metabolized tracer, and the cytokine content in plasma and CSF during postnatal development in normobilirubinemic rats in order to increase our knowledge on these parameters which remain little characterized during development. In particular, developmental concentration profiles of most cytokines in CSF are currently unknown. In parallel, we examined whether these three parameters are modified in hyperbilirubinemic Gunn animals. We also assessed whether the neuroinflammatory response of the choroid plexus-CSF system to a bacterial stimulus was exacerbated in hyperbilirubinemic newborn rats. Altogether, the data point out important early postnatal functional changes in the blood–brain interfaces/CSF system, and show that among brain cells, those forming the blood–brain interfaces do not appear to be primary targets of bilirubin toxicity.

## Material and methods

### Animals, genotyping, fluid and tissue sampling

Gunn rats were obtained from the SPF animal facility of the University of Trieste and the colony was maintained at the Lyon Neurosciences Research Center animal facility. Animal homozygous for the mutation (jj genotype) were characterized by a yellow skin color clearly visualized shortly after birth, in contrast to wildtype animals (NN genotype) and animals bearing the heterozygous mutation (Nj genotype). They also developed a mild cerebellar hypoplasia (Additional file 1: Fig. S1). Total serum bilirubin concentration strongly increased to 11 mg/dl after birth, and kept increasing up to 15 mg/dl (257 µM) 17 days after birth in jj animals (Additional file 1: Fig. S1). We previously reported the calculated free bilirubin concentration in the blood of jj animals, which was 154 nM in 2-day-old rats, was maintained at a high value (117 nM) in 9-day-old animals, and decreased thereafter with the parallel increase in albumin concentration [21]. These high bilirubin levels observed in the early postnatal stages correspond to severe hyperbilirubinemia necessitating phototherapy or exchange transfusion in human babies [23]. In contrast, Total serum bilirubin levels measured in Nj animals were similar or only slightly above control levels in the early postnatal stages (Additional file 1: Fig. S1), and the calculated free bilirubin concentration did not exceed 1 nM [21]. In jj animals, bilirubin diffused into the central nervous system, as shown by the yellow color of the CSF and brain tissue. The genotyping needed to differentiate wildtype (NN) from heterozygous (Nj) animals was performed by PCR using the forward primer 5′-GGG ATT CTC AGA ATC TAG ACA TTG T-3′ and the reverse primer 5′-TCG TTT GTT CTT TTC TAT TAC TGA CC-3′ (detailed conditions in Additional file 1: Fig. S1). The amplicon was then digested for 3 h at 60 °C with BstN1 (NEB BioLabs), whose consensus sequence is suppressed by the single mutation deletion in the mutated gene. Gel electrophoresis allows to discriminate samples from NN animals (2 bands: 231 and 80 bp) from Nj animals (3 bands: 311, 231 and 80 bp) (Additional file 1: Fig. S1).

For immunohistochemistry, cytokine measurements, and CSF leukocyte counts, fluid and tissue were sampled as follows: animals were injected intraperitoneally with pentobarbital (Euthasol, 0.34 (1 or 2-day-old), 0.2 (9-day-old), 0.1 (17-day-old) mg of pentobarbital/g as an 1/8 dilution in saline), blood was withdrawn on heparin by intracardiac puncture, and the cisterna magna was exposed. CSF was sampled using a glass micropipette, collected in a low binding tube, and its volume measured. Heparinized blood samples were centrifuged at 5000 rpm and plasma were collected. Fluids were frozen at − 80 °C until use. Following fluid sampling, the brains were dissected out of the cranial box, quickly frozen in isopentane at − 45 °C, and stored at − 80 °C until used for immunohistochemistry.

### Permeability measurements

The permeability of the blood–brain barrier (at the levels of cortex, cerebellum, pons) and of the blood–CSF barrier was measured towards [^14^C]-sucrose used as a polar marker of the barrier cell integrity. The influx constants, apparent brain K_in_ (App K_in_) and true CSF K_in_ for [^14^C]-sucrose were determined as described previously [24, 25]. Briefly, [^14^C]-sucrose was injected intraperitoneally to animals under slight isoflurane anesthesia. Plasma and CSF were sampled after 20 min as described above. Their radioactive content was measured by liquid scintillation counting in a TRICARB 4910 TR (Packard). The brain was collected and frozen on dry ice. Pieces of cortex, pons, and cerebellum, free of meninges, were dissected from the frozen brain in a − 20 °C chest freezer, weighted, and digested in 1 M sodium hydroxide overnight at 4 °C. The digested tissues were further homogenized with a micro-pestle before being analyzed for radioactive content. [^14^C]-Sucrose plasma concentration x time area-under-the-curves (AUCs) from 0 to 20 min (AUC _0→20 min_) were calculated for each individual [^14^C]-sucrose plasma concentration at 20 min, using the shape of a generic time x sucrose plasma concentration curve obtained by the litter-based method on animals sacrificed at various time points between 2 and 30 min. As no differences were found in sucrose plasma concentrations over time between males and females, the values were pooled to build up the generic sucrose plasma concentration x time curves for 1-day-old and 9-day-old animals. The curves were built separately for NN/Nj and for jj animals, as the jj genotype slightly affected the shape of the curves at both ages (Additional file 1: Fig. S2). K_*in*_ values were calculated using the following equation:

$${\text{K}}_{{{\text{in}}\,csf}} = \, [ {^{{{14}}} {\text{C}}} ]\text{-} {\text{sucrose C}}_{{{2}0{\text{min}}}} /{\text{AUC}}_{{0 \to {2}0{\text{min}}}} ,$$

where C_20min_ is [^14^C]-sucrose concentration in CSF at 20 min, expressed as µl . ml (of csf)^−1^. min^−1^, i.e. 10^–3^ min^−1^.

$${\text{App K}}_{{{\text{in}}\,brain}} = \, [ {^{{{14}}} {\text{C}}} ] \text{-} {\text{sucrose C}}_{{{2}0{\text{min}}}} /{\text{AUC}}_{{0 \to {2}0{\text{min}}}} ,$$

where C_20min_ is [^14^C]-sucrose concentration in discrete brain regions at 20 min, expressed as µl . g^−1^ . min^−1^.

For 9-day-old animals, App K_in *brain*_ values were corrected for the amount of sucrose associated to the brain vascular space to generate true K_in *brain*_, using the following plasma volume determined as described infra: Cerebral cortex: 5.0 ± 1.4, Pons: 5.7 ± 0.7, Cerebellum 12.6 ± 0.6 µl/g, (mean ± SEM, n = 3 to 6 from 3 different litters).

### Plasma volume measurement in brain tissue

After pentobarbital anesthesia, a blood sample was collected by intracardiac puncture and plasma was frozen. Half of the animals from one litter underwent a transcardiac perfusion with a physiological solution (Hank’s balanced salt solution supplemented with 10,000 U/l heparin. Perfusion was performed at a rate of 0.75 ml/min for 20 min in newborn animals, 1.4 ml/min for 15 min in 9-day-old animals, and 3 ml/min for 12 min in 18-day-old animals. Brains from perfused and non-perfused animals were collected and frozen on dry ice. Pieces of cerebral tissue, free of meninges, were dissected out of the frozen brain in a − 20 °C chest freezer, weighted, and homogenized in phosphate buffer saline (PBS; in mM: NaCl 150; Na_2_HPO_4_ 12; KH_2_PO_4_ 2; pH 7.4) (5 s sonication, amplitude 50%, UP50H Hielscher sonicator). Rat IgG concentrations were measured in plasma and brain homogenate samples by ELISA (Bethyl Laboratories, E110-128 Kit). The vascular volume, expressed as µl/g, was calculated as being the difference of the IgG amount per gram of tissue between non-perfused and perfused animals, divided by the IgG plasma concentration in the non-perfused animals.

### Analysis of the neurovascular network

Rat brains were cut at − 20 °C using a NX50 Microm Microtech cryostat. Ten-µm thick sagittal sections were collected on glass slides and stored at − 20 °C until used for immunostaining. Sections were fixed at room temperature in 4% paraformaldehyde in PBS for 10 min, then blocked for 1 h in 1% BSA, 8% normal goat serum, 0.3% Triton in PBS. After overnight incubation at 4 °C with the anti- RECA-1 antibody (Bio-rad, MCA970GA, 1.7 µg/ml) in PBS supplemented with 1% BSA, 1% normal goat serum, and 0.3% Triton, sections were washed 3 times with PBS supplemented with 0.3% Triton, and incubated with secondary Alexa Fluor 488-conjugated goat antirabbit antibodies (Invitrogen, Carlsbad, CA; 2 µg/ml) for 1 h at room temperature. Sections were counterstained with DAPI for 10 min and then mounted in aqueous medium. Negative controls were performed by omitting the first antibody. For each area of analysis, three serial sections were examined at X160 with a fluorescence stereo zoom microscope (Zeiss Axio Zoom.V16) equipped with an AxioCam 503 camera, and image acquired through zen 2.3 Pro software (Zeiss Microscopy). Quantitative analysis was done using ImageJ software to determine the number of vessel segments per field as well as the vessel surface area (µm^2^). Areas with large vessels were omitted from the study by introducing a filter in the analysis that takes into account only vessel profiles whose surface area were comprised between 10 and 25,000 µm^2^. For each animal and each area of analysis, data from the 3 serial sections were averaged. The number of microvessel segments per field of observation and the vessel area per field of observation were used as proxies for the quantification of microvascular network development and vascular volume, respectively.

### Plasma and CSF cytokine content

Cytokines, including several chemokines were analyzed by use of a MILLIPLEX® MAP Rat Cytokine/Chemokine Magnetic Bead Panel RECYTMAG-65 K (Millipore) according to the manufacturer’s protocol. Sample volume needed for plasma and CSF analyses, in singlicate, at the minimal required dilution recommended by the manufacturer (ie 2), was 15 µl. When CSF sample withdrawn from one animal was smaller than 15 µl, samples from 2 animals were pooled, and plasma of these two animals were pooled in the same proportions. Analyte quantification was performed on the Bioplex-200 platform.

### Assessment of leukocyte infiltration in CSF

Eight-day-old rats were injected intraperitoneally with 1 mg/kg PAM3CSK4 (P3C, Merck Millipore France), or 0.9% saline. CSF was sampled 14 h later [5], and total leukocytes and polymorphonuclear neutrophiles (PMNs) were counted in a Bürker chamber after staining with Türk’s solution (Sigma-Aldrich). The phenotypic analysis of whole blood immune cells was performed by immunocytochemistry following red blood cell lysis as previously described [26].

### Statistical analysis

Statistical tests and significance are described in figure legends when appropriate.

## Results

### Neurovascular network development

**Fig. 1:**
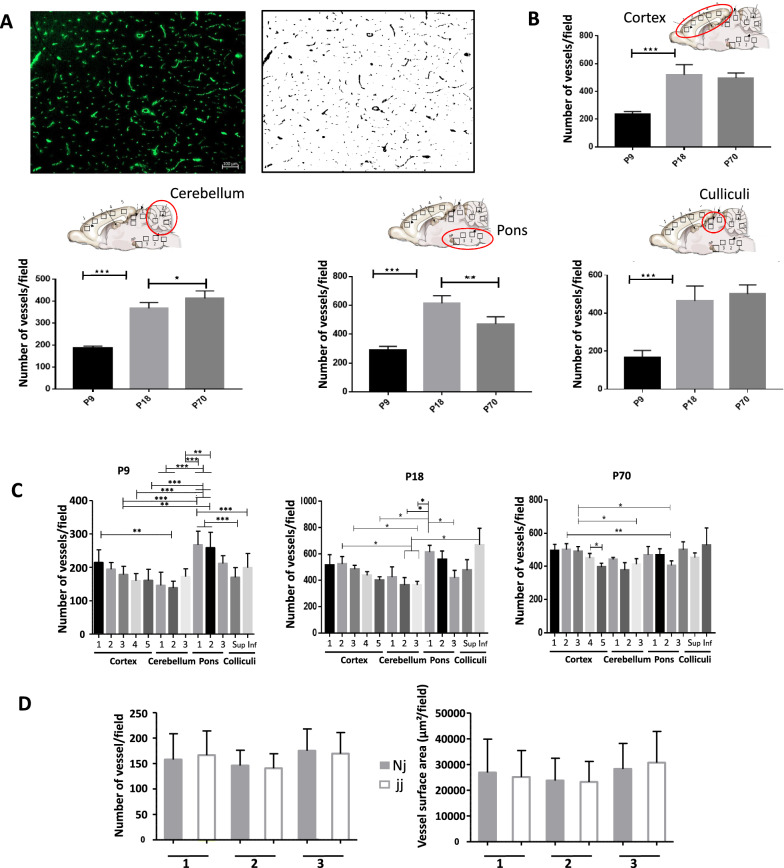
Cerebral vascularization in normobilirubinemic and hyperbilirubinemic animals during post-natal development. **A** Typical immunohistological staining of the endothelial-specific RECA-1 protein on brain section, and corresponding transformed black and white image generated using ImageJ software and allowing to quantify the number of microvessel segments and the surface area per field of observation (1.42 mm^2^ field); **B** Number of vessel segments per field in the cortex, cerebellum, pons and colliculi at three postnatal developmental stages in normobilirubinemic Nj animals. The data represent an average of measurements from 3 to 5 different locations (squares on the drawings) within each structure; **C** Comparative analysis of the number of vessel segments in several subregions of brain structures at the three different ages studied in normobilirubinemic Nj animals; **D** Comparative analysis of the number of vessel segments (left), and of vessel surface area (right), per field in three regions of the cerebellum (insert in **B**) between non-jaundiced and jaundiced 9-day-old animals. All data are expressed as means ± SD n = 4 animals (2 males, 2 females) for 9-day-old (P9) and 70-day-old rats (P70), and n = 6 animals (3 males, 3 females) for 18-day-old rats (P18), for each genotype. All fields had the same surface area of 1.42 mm^2^. *, **, ***: statistically different values, p < 0.05, p < 0.01, p < 0.001, respectively, Anova followed by Tukey’s multiple comparisons test. For 9-day-old animals in **C**, only ** and *** significances are shown

Cerebral endothelial cell proliferation leads to prominent extension and growing complexity of the vascular network postnatally [27]. To test whether the early postnatal rise in plasma unconjugated bilirubin alters the development of the vascular network, we analyzed the number of vessel segments and the vessel surface (Fig. 1A) in different areas of the cerebral cortex, pons, cerebellum, and in the inferior and superior colliculi, using histological sections of brain from normobilirubinemic Nj and hyperbilirubinemic jj animals after extended periods of jj animal exposure to hyperbilirubinemia, i.e. on postnatal day 9 and 18, and in adult. The extent of the vascular network (Fig. 1B) and vascular volume (not shown) strongly increased from postnatal day 9 to 18 in all brain regions of healthy rats. It remained steady thereafter, except in the pons where the vessel density decreased in adult as compared to 18-day-old animals, possibly as a result of myelination in this white matter area (Fig. 1B). The data also highlighted regional variations in the complexity of the cerebral vasculature in 9-day-old animals, such as a less developed vascular network in cerebellar and occipital cortices than in frontal cortex, and a higher level of vascularization in the pons than in other structures. These variations were less prominent or disappeared in later stages (Fig. 1C). When comparing the vascular network between normal animals and animals with hyperbilirubinemia at all three developmental stages, no difference in the extent of the vascular network (Additional file 1: Fig. S3), or in the vascular surface area (not shown) was observed on postnatal day 9 or at later investigated stages. This held true even in the cerebellum (Fig. 1D) a structure whose growth is mainly postnatal, and whose size is reduced by hyperbilirubinemia in the Gunn rat ([22] and Additional file 1: Fig. S1).

### Integrity of the barriers

**Fig. 2:**
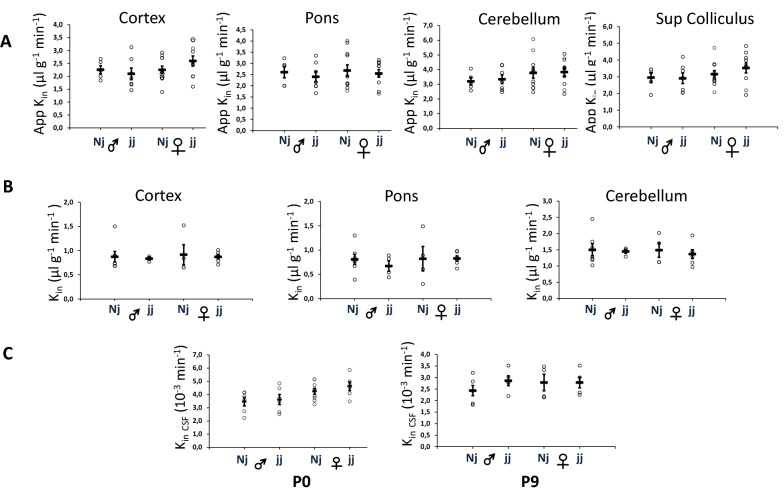
Blood–brain and blood–CSF barrier integrity in normobilirubinemic and hyperbilirubinemic male and female rats during post-natal development. The integrity of the barriers was assessed by measuring their permeability toward [^14^C]-sucrose used as a polar tracer. **A** Apparent Kin brain measured in 8-h-old animals. **B** True K_in brain_ measured in 9-day-old animals. **C** K_in CSF_ measured in 8-h-old (P0) and 9-day-old (P9) animals. Data are expressed as means ± SEM, individual values are shown as open circles. Note that P0 and P9 tissue data are expressed as apparent and true K_in_, respectively. See methods for permeability constant definition and calculation

To assess whether an alteration in the integrity of either the blood–brain or blood–CSF barrier is triggered by an immediate or sustained increase in free plasma bilirubin levels, we compared the blood-to-CNS transfer of sucrose, a small inert low-permeant molecule, between non-jaundiced and jaundiced animals. Analyses were done in three different brain regions and in CSF, both in 8-h-old and 9-day-old animals. These two time points reflect an acute (few hours) and sustained (9 days) barrier cell exposure to bilirubin, respectively. For 9-day-old animals, true brain tissue K_in_ values were obtained by correcting apparent K_in_ values for the amount of sucrose remaining in the brain vascular space (see methods). This correction could not be made for 8-h-old animals, as the low difference in cerebral IgG levels between perfused and non-perfused animals prevented an accurate determination of the vascular volume at that stage. The blood–brain barrier permeability toward sucrose was not different between male and female normobilirubinemic rats (Fig. 2A, B). As only apparent K_in_ values were generated in 8-h-old animals, age-dependent permeability changes could only be assessed by comparing apparent K_in_ in tissue. The mean apparent K_in_ (in µl g^−1^ min^−1^) decreased from 2.34 to 1.14 in the cortex, from 3.26 to 1.77 in cerebellum, and from 2.63 to 1.43 in the pons between 8-h-old and 9-day old animals (p < 0.001, bilateral student-t test for unequal variance), despite the concurrent growth of the vascular network in all these structures. Similarly, the blood–CSF barrier permeability to sucrose did not differ between male and female (Fig. 2C) and decreased from 3.84 to 2.57 10^–3^ min^−1^ between 8-h-old and 9-day-old animals (p < 0.01, bilateral student-t test for unequal variance, Fig. 2C). Finally, no difference was observed in the blood–CSF or blood–brain barrier sucrose permeability constants between hyperbilirubinemic and normobilirubinemic animals at both ages (Fig. 2).

### CSF and plasma cytokine content

27 Cytokines including 5 chemokines were investigated by the multiplexed antibody-based technology in the CSF and plasma of NN, Nj and jj animals.

There was no difference between males and females in the levels of immune mediators in either the plasma or the CSF at all stages of development investigated and in any of the genotype groups. Data from both sexes were therefore pooled in the following analyses.

**Fig. 3:**
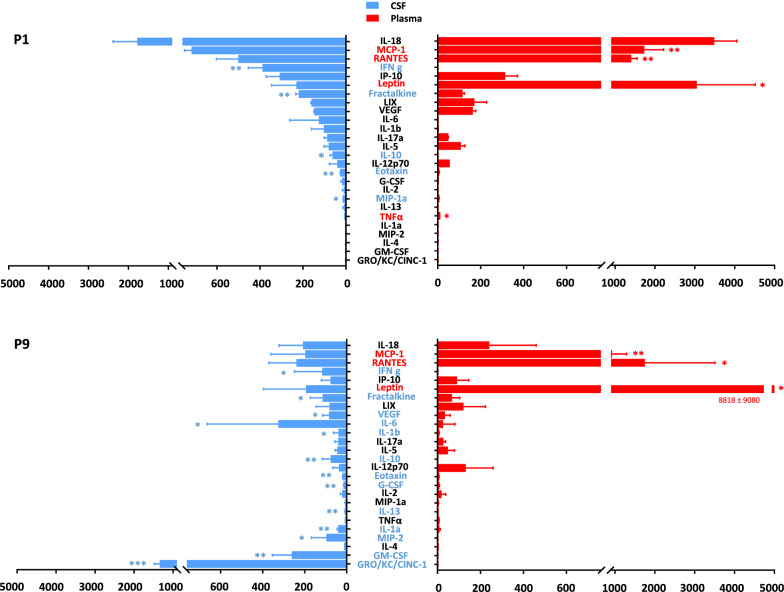
Comparative analysis of cytokine levels in CSF and plasma of 1-day-old and 9-day-old wild-type animals. Data are expressed as pg/ml, mean ± SD of 4 and 8 samples from 1- and 9-day-old animals, respectively. *, **, ***: adjusted p < 0.05, p < 0.01, p < 0.001, respectively, paired t-test adjusted for multiple comparisons by controlling the False Discovery Rate set at 0.05. Cytokines whose mean did not exceed the lower limit of quantification in any group (Il-4, EGF) were not included in the statistical analysis. P1, P9: 1-, 9-day-old animals, respectively

**Fig. 4:**
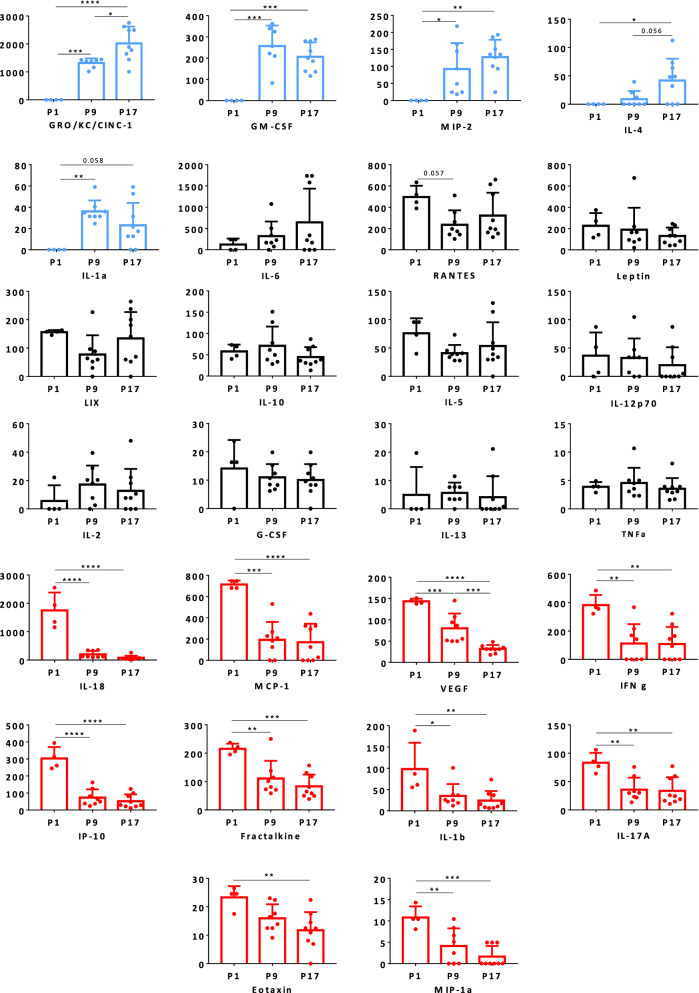
Developmental profiles of cytokines in the CSF of wild-type animals. Data are expressed as pg/ml (mean ± SD of 4, 8 and 9 samples from 1-, 9- and 17-day-old animals, respectively). *, **, ***: p < 0.05, p < 0.01, p < 0.001, respectively, Anova followed by Tukey’s multiple comparisons test. P values are indicated also for differences close to 0.05 significance. EGF was not detected at any stages. P1, P9, P17: 1-, 9-, 17-day-old animals, respectively. Genes depicted in blue are developmentally upregulated genes, those depicted in red are developmentally downregulated genes

The global comparison of cytokine levels between CSF and plasma of normobilirubinemic (NN) animals in 1-, 9- and 17-day-old animals is shown in Additional file 2: Fig. S4. It revealed two types of information. First, the profile of cytokines ranking in term of abundance in the CSF differs from that in the plasma, at the three stages investigated (Fig. 3, and Additional file 2: Fig. S4). For instance, in 1-day-old NN animals, leptin is abundant and much higher in the plasma than in CSF, while IFNγ is abundant in CSF, but absent in plasma. High levels of IL-18, RANTESs, MCP-1, IP-10 are found both in CSF and plasma, RANTES and MCP-1 displaying higher concentration in plasma than in CSF (Fig. 3). In 9-day-old and 17-day-old animals, leptin, RANTES and MCP-1 are the most abundant cytokines found in the plasma, with concentration exceeding those found in the CSF, while CINC-1, is the most abundant cytokine found in the CSF and is not detected in the plasma. The cytokines GM-CSF and MIP-2, IL-10 and IFNγ are also found in CSF but not in plasma (Fig. 3). Second, the CSF and plasma profiles of abundance observed in 1-day-old animals differ from that of 9- and 17-day-old animals. For example, high levels of IL-18 are observed in the CSF and plasma of 1-day-old animals, but not at later stages. CINC-1 and GM-CSF are absent in the CSF of 1-day-old animals, but detected at high levels in the later stages (Additional file 2: Fig. S4, red arrows). We therefore represented the CSF and plasma developmental profiles obtained from NN animals for each cytokine. In CSF of 1-day-old animals, CINC-1, IL-6, GM-CSF, MIP-2, IL-4 and IL-1α were undetected or present at low levels, and increased thereafter. In contrast, levels of IL-18, MCP-1, vascular endothelial growth factor (VEGF), IFNγ, IP-10, fractalkine, IL-1β, IL-17α, eotaxin and MIP-1α were high in the CSF of 1-day-old animals as compared to 9- or 17-day-old animals (Fig. 4). In plasma, the interindividual variability was higher than in CSF. Developmental variations were restricted to IL-1β present at a low level in 1-day-old animals as compared to levels measured at later stages of development, and to IL-18, MCP-1, IP-10, VEGF whose levels were higher in 1-day-old animals than in 9- or 17-day-old animals. (Additional file 2: Fig. S5).

**Fig. 5:**
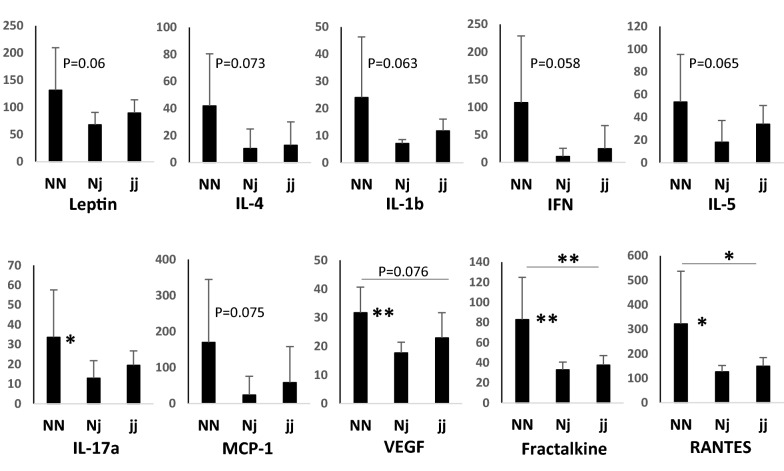
CSF cytokine content in normobilirubinemic and hyperbilirubinemic seventeen-day-old animals. Only cytokines for which differences between genotypes were statistically significant or close to significance are shown. Data are expressed as pg/ml, (Mean ± SD from 9 samples). *, **: p < 0.05 and p < 0.01, respectively, Anova followed by Tukey’s multiple comparisons test

The comparative analysis of normo- and hyperbilirubinemic animals revealed no difference in the CSF or plasma levels of cytokines between NN and jj animals in 1-day-old rats (i.e. shortly after the free bilirubin plasma level increases in jj rat pups), (not shown). The differences closest to statistical significance were observed for CSF levels of IL-1β (p < 0.077) and RANTES (p < 0.053), both being lower in jj than in NN animals. In 9-day-old rats that underwent (when carrying the jj genotype) sustained exposure to bilirubin, no difference in cytokine concentration was observed in CSF between NN, Nj and jj animals (not shown). In the plasma of these animals, only the level of MCP-1 was significantly but slightly (1.3 fold) increased in Nj animals as compared to NN animals. This increase was not observed in hyperbilirubinemic jj animals (Anova followed by Tukey multiple comparisons test). In 17-day-old animals, changes in cytokine levels between animal groups were also limited, both in CSF and in plasma. In CSF, Nj animals had overall decreased levels of cytokines by comparison to NN animals, as exemplified in Fig. 5 for polypeptides whose concentration differences reached, or were close to statistical significance. This decrease was less marked but also observed in jj animals. In plasma of these 17-day-old animals, a trend to lower concentrations of selected cytokines was also observed in Nj animals by comparison to NN animals (Additional file 2: Fig. S6). This was not observed in jj animals in which cytokine levels were similar to those measured in NN animals. To validate our multiplex approach to detect an inflammation in the CSF, we treated P8 Sprague–Dawley rats with P3C, a TLR-2 ligand lipopeptide derived from gram negative bacteria, that induces CSF inflammation in developing animals [5]. CSF samples of these animals were used as positive controls for CSF inflammation and were analyzed simultaneously with samples of normobilirubinemic and hyperbilirubinemic animals. Following P3C treatment, we found a dramatic increase (from 3 folds to 3 orders of magnitude) in the CSF level of IL-1α, IL-1β, IL-6, IFNγ, IL-5, TNFα, MCP-1, IP-10, fractalkine, LIX, G-CSF, resulting in concentrations higher than 1 ng/ml for IL-6, MCP-1, IP-10. We also found a strong increase in the plasma levels of MCP-1, IP-10, fractalkine, and RANTES in these animals (data not shown).

### Blood–CSF infiltration of leukocytes following bacterial lipopeptide exposure

**Fig. 6:**
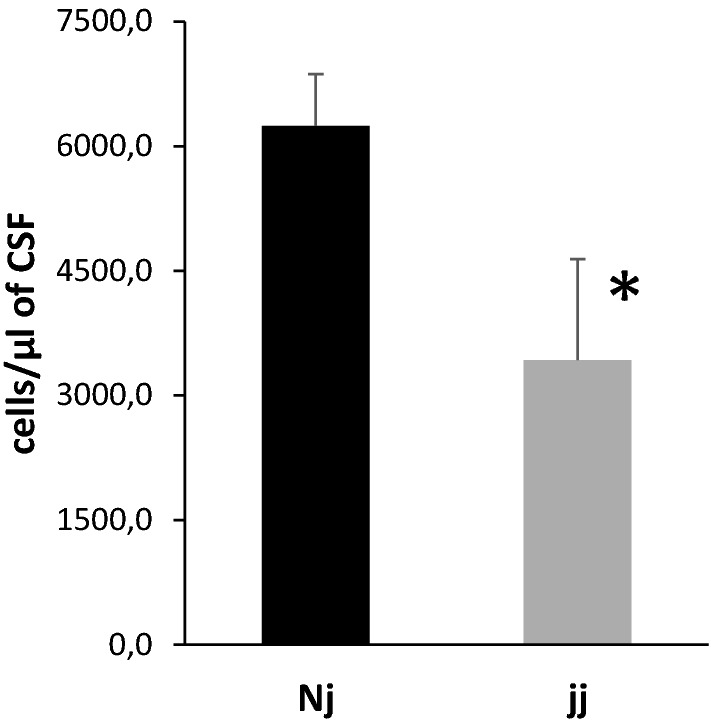
Neutrophile infiltration in CSF induced by P3C treatment in normobilirubinemic and hyperbilirubinemic developing rats. P3C was injected in 8-day-old animals and CSF sampled 14 h later. P3C induced a massive pleocytosis, and 91% of immune cells found in the CSF of Nj animals were PMNs. The pleocytosis was reduced in jj rats. Data are expressed as the number of PMNs counted in 1 µl of CSF, mean ± SEM, n = 9 (Nj) and 11 (jj) from 3 jj and 2 Nj litters. *, p < 0.05, one-tailed student’s t-test for unequal variance

Systemic exposure of developing animals to the gram negative bacterial lipopeptide P3C leads to an infiltration of white blood cells into the CSF through the choroid plexuses [5]. To assess whether the hyperbilirubinemic pups were more prone to neuroinflammation, we injected P3C in 8-day-old hyperbilirubinemic jj and normobilirubinemic Nj animals issued from three mixed litters, and in age-matched NN animals originating from independent litters. As expected, an important pleocytosis was observed in Nj rats (Fig. 6) and NN animals (not shown). A large majority (91%) of the cells found in CSF in these animals were PMNs. The percentage of white blood cells identified as PMN in the blood of jj animals was not different from that measured in Nj animals from the same litters (36,0 ± 3,0 versus 36,0 ± 1,2, respectively, mean ± SD), and exposure of jj animals to P3C led to a 46% increase in PMNs circulating in the blood, as observed in a normobilirubic animal response (data not shown). Yet the number of PMNs infiltrating the CSF in jj rats was half that observed in Nj animals, indicating that CSF inflammation induced by a systemic bacterial stimulus is substantially attenuated by the jj genotype.

## Discussion

This work first brings forwards developmental specificities of the brain vasculature and shows that the latter is not impacted by hyperbilirubinemia. The analysis of the number of vessel segments and of the vessel surface per surface area of thin brain sections provides an index of the microvascular network complexity and volume, respectively. It shows that the parenchymal microvascular network undergoes extensive growing complexity between 9 and 18 days after birth in the cerebral cortex of rodents as previously described in pioneer studies [27, 28] and more recent works based on refined imaging analysis [29–32]. The data also show that this growing complexity extends to other brain areas such as the cerebellum and midbrain, and reveals a region-to-region heterogeneity in the vascular network development during the early postnatal period, that is levelled by day 18.

The cerebral capillaries have both scaffold and paracrine signaling functions that modulate the development and differentiation of multiple neural cell populations, including those migrating radially from neurogenic niches to cortical areas [3, 33]. Conversely, a change in neural activity affects postnatal angiogenesis. Reducing sensory input decreases the cortical vascular density and branching, while an enhancement of neural activity leads to different types of vascular alterations according to different studies [30, 34]. Both the formation of cerebral vessels and the postnatal cerebral blood flow which increases drastically in parallel with the neurovascular network between postnatal day 10 and 20, are sensitive to oxidative stress such as induced by hypoxia [29, 35]. Given these complex interactions between neuronal activity, vascular network development, and cerebral blood flow, and considering that bilirubin induces neuronal toxicity and generates oxidative stress, we hypothesized that the development of the postnatal vascular network would be altered in animals with pathological hyperbilirubinemia. We did not observe any effect during vasculogenesis on postnatal day 9, nor after vasculogenesis on postnatal day 18 and in adult. This holds true even in the cerebellum whose postnatal growth is strongly impacted by hyperbilirubinemia (this paper, [22]). These data indicate that the mechanisms linking the vascularization to the overall growth of brain structures are maintained in hyperbilirubinemic animals, and suggest that oxygen supply to the brain during postnatal development is not impacted by chronic exposure to bilirubin. Whether the growth of the cerebellum in jj animals adapts to the development of the vasculature, or the vasculature adapts to the reduced cerebellar growth remains to be understood.

The blood–brain permeability to sucrose measured in different brain regions is not altered by bilirubin either, whether sucrose transfer across the blood–brain barrier is measured a few hours after bilirubin concentration rises in the blood, or following a chronic exposure of endothelial cells to bilirubin, such as in 9-day-old rats. Similarly, no change in the blood–CSF permeability to sucrose is observed following choroidal exposure to high plasma concentrations of bilirubin. Barrier permeabilities were evaluated by measuring sucrose k_in_ constants which represent the fraction of the blood-borne molecule that crosses barriers to reach the CSF and neuropil. The distribution of polar tracers such as sucrose into the CSF and brain depends on the efficacy of tight junctions to seal the paracellular pathway between blood and brain/CSF compartments, the formation of transcytotic pathways across barriers cells, the surface area available for exchange, the rate of CSF-extracellular fluid exchange, the brain-to-blood backflux, and the CSF turnover rate. The impact of the last two parameters is minimized in our 20-min experimental setting. Endothelial and choroidal junctions are already tight at birth [1, 36], but may be more fragile when facing a pathophysiologic, or toxicologic stress. Together with the formation of tight junctions, non-specific endothelial transcytosis decreases rapidly during development under the influence of pericytes [37, 38]. Overall this explains the efficiency of blood–brain interfaces to prevent the movement of polar compounds from blood to the developing brain. Still, higher k_in_ values towards sucrose have been measured in 1-day-old rats as compared to 9-day-old animals, not only in CSF but also in tissue despite the less developed vascular network in 1-day-old animals. The reasons for this age-related decrease in apparent permeability are not fully elucidated and have been discussed elsewhere [24, 36, 39]. While in 1-day-old rats, part of the microvessels are not perfused, most (> 90%) of them are perfused in 9-day-old animals [40]. As the extent of the vascular network does not change in hyperbilirubinemic animals at either stage of development (Fig. 1), the similar sucrose permeability measured in normo- and hyperbilirubinemic animals indicates that the integrity of the blood–brain barrier is not impacted by bilirubin, and suggests that bilirubin does not affect the extent of vessel perfusion in early postnatal life either. The preservation of the choroidal blood–CSF barrier in hyperbilirubinemic animals is corroborated by the maintenance of the barrier integrity in a cellular model formed by a tight monolayer of differentiated choroid plexus epithelial cells in primary culture, chronically exposed to a pathophysiologically relevant concentration of bilirubin [41].

Alteration of the blood–brain barrier involving tight junction disorganization has been reported at the adult stage in animal models of hepatic encephalopathy in which serum bilirubin levels are abnormally high [42–44]. The data however point to the implication of noxious agents other than bilirubin in this alteration. Such agents could be ammonia, or selected inflammatory cytokines released in the plasma as a result of liver failure. No imaging studies in babies investigated the influence of a pathological postnatal rise in plasma bilirubin concentration on the integrity of blood–brain barriers. One postmortem analysis reported an increased vascularization and signs of tight junction alterations, possibly linked to VEGF signaling, in the brain of a baby born prematurely with kernicterus associated with signs of severe hypoxia [45]. Our histological and functional data indicate that bilirubin itself is unlikely to be responsible for these alterations, especially as confounding factors such as hypoxia and ischemia are known to activate VEGF signaling. This is in line with an experimental study coupling hypoxia-induced acidosis with injection of bilirubin in 3-day-old mice. An alteration of the blood–brain barrier was observed in these animals, that resulted from acidosis, and not from bilirubin exposure [46]. Finally, Roger et al.found that bilirubin entered the brain without any sign of blood–brain barrier alteration following a two-hour intravenous infusion of bilirubin at a pathological concentration in 10-day-old rats [47]. The brain barriers harbor numerous transporters, among which neuroprotective ABC transporters, whose expression is developmentally regulated [48]. Evidence that unconjugated bilirubin alters the expression of some of these transporters during postnatal development and in adult has been brought forwards [49], and reviewed [50, 51]. Collectively, our data collected in the Gunn rat which brain is exposed to endogenous plasma bilirubin at a pathologically relevant concentration, and the literature based on exogenously injected bilirubin, indicate that search for direct effects of bilirubin on brain barriers should be oriented towards changes in transport functions rather than an overt impairment of the barrier integrity.

The choroid plexuses are rapidly activated following systemic inflammation and the CSF supports pro-inflammatory mediator circulation [4]. We detected a number of immune mediators in the CSF of normobilirubinemic animals. Our data show that in developing animals cytokine profiles in CSF and plasma are not correlated. In plasma the most abundant cytokines include RANTES and MCP-1 as observed in human newborns [52]. The source of most cytokines found in plasma is likely to be circulating immune cells. In CSF the data also unravel different kinetics of cytokines during development. For instance, CSF levels of CINC-1/CXCL1, IL-6, GM-CSF, MIP-2/CXCL2, IL-4 and IL-1α were low in 1-day-old animals and increased strongly thereafter, while the opposite was observed for IL-18, MCP-1/CCL2, VEGF, IFNγ, IP-10/CXCL10, fractalkine/CX3CL1, IL-1β, IL-17α, eotaxin/CCL11, MIP-1α/CCL3. These data suggest that these immune mediators fulfill brain-specific physiological functions during development, independent of their functions as inflammatory modulators. Indeed, besides VEGF whose function is well understood in neurovascular development [53], cytokines classically associated with the immune system are also involved in neuronal differentiation/migration, synaptic plasticity, and neuroendocrine organization during development [6, 54, 55]. As examples CINC-1 induces the proliferation and limits the migration of oligodendrocyte progenitors [56]. Fractalkine also promotes oligodendrogenesis [57] and controls microglial functions necessary for early postnatal brain maturation [58]. Indirect evidence based on intrauterine growth restriction associated with IL-4 overproduction indicates that IL-4 regulates oligodendrogenesis during postnatal development [59]. IL-6 is involved in cortical white matter development, motor development, and shapes long-term social behavior [60], hence participating in the development of autistic syndromes [61]. IFNγ acts as an efficient negative regulator of neural precursor cell activity and differentiation into neurons in adult [62], a function that remains to be explored in the context of postnatal development.

To our knowledge our study is the first to report specific CSF developmental profiles for these immune cytokines in the healthy rat during the postnatal period. The physiological meaning of the changes in CSF concentration for each individual cytokine needs to be investigated, as is their cellular origin. The choroid plexus epithelial cells, ependymal cells including tanycytes and immune and non-immune cells harbored in the ventricular, subarachnoid, and cisternal spaces are potential sources, in line with the role attributed to the choroid plexus-CSF system in securing brain development [4, 63]. Astrocytes and microglial cells found in the periventricular neurogenic niches, and even neurons located deeper in the brain parenchyma are also potential sources of cytokines circulating in CSF [56, 64]. Transporters for selected cytokines have been described at blood–brain interfaces [65]. However, plasma is unlikely to be the main source of CSF cytokines, because developmental changes observed in plasma did not overlap those observed in CSF.

Considering the proximity of CSF compartments with periventricular and hippocampal stem cell niches which harbor the main cellular targets of cytokines during development, CSF-borne cytokines are likely to fulfill important endocrine signaling. An alteration of the developmental profile of these bioactive molecules in CSF, induced by perinatal injuries such as sepsis, may not only participate in spreading systemic inflammation to the brain, but also directly lead to unbalanced neuronal signaling. This would ultimately alter normal neural network organization and lead to neurodevelopmental diseases [6, 66, 67]. As bilirubin impacts only marginally CSF cytokine content, it is unlikely that hyperbilirubinemia-induced cerebral dysfunctions involve a similar pathophysiological mechanism. Of note the somewhat large standard errors that can be attributed to both inter- and intra-litter variability, and to the analytical performances of the multiplex method, have limited the statistical power of the study, which would deserve further investigation by analyzing selected cytokines such as those listed in Fig. 5 with more sensitive methods. The cytokine concentrations measured in CSF of NN rats tend to be higher than concentrations found for Nj rats with a mild physiological hyperbilirubinemia, or jj rats presenting high pathological levels of free bilirubin in plasma. This effect could be related to the systemic anti-inflammatory, or immunosuppressive properties attributed to bilirubin [68–70], independently of its toxic effect on neural cells. In line with this we also observed a decrease in innate immune cell infiltration in the CSF of hyperbilirubinemic as compared to normobilirubinemic animals following systemic exposure to a gram negative bacteria lipopeptide. Whether this reflects a direct impact of bilirubin on circulating innate immune cells, especially PMNs, or a change in the choroidal attributes that set the migration of these cells into the CSF remains to be elucidated. Altogether these data suggest that hyperbilirubinemia does not activate choroid plexus and does not induce an important inflammatory response in CSF that could trigger the neurological impairment observed in pathological jaundice.

## Conclusions

In conclusion we describe postnatal developmental changes in the vascular network formation and in blood–brain interfaces permeability, and show that none of these parameters is impacted by acute or chronic hyperbilirubinemia mimicking the human postnatal pathologic jaundice. Our study also provides evidence for a central and a peripheral pool of cytokines that are of different origins and a developmental regulation of several CSF-borne cytokines, that suggest a specific role of selected cytokines in neurodevelopment. The influence of hyperbilirubinemia on both CSF and plasma cytokines is marginal, and bilirubin reduces innate immune cell infiltration in the brain of inflamed animals. Altogether our data indicate that a direct effect of bilirubin on the vascular system organization and brain barriers integrity, and the inflammatory response of the choroid plexus-CSF system to bilirubin, are not involved in the alteration of brain maturation induced by neonatal jaundice.

## Supplementary Information

**Additional file 1. **Additional figures S1, S2 and S3.

**Additional file 2.** Additional figures S4, S5 and S6.

## Data Availability

Data generated or analyzed during this study are included in this published article and its additional files.

## Abbreviations

AUC: Area-under-the-curve
P3C: PAM3CSK4 lipopeptide
PBS: Phosphate buffer saline
PMN: Polymorphonuclear neutrophile
