# Supplementary material for: Vascular network expansion, integrity of blood–brain interfaces, and cerebrospinal fluid cytokine concentration during postnatal development in the normal and jaundiced rat
Source: Fluids Barriers CNS. 2022 Jun 7;19:47. doi: 10.1186/s12987-022-00332-0 (PMC9172137; doi:10.1186/s12987-022-00332-0)

Supplementary Figure 1. Characteristics of the Gunn rats used for the study. A, animals homozygous for the mutation (jj phenotype) were characterized by a yellow skin color illustrated here in seven-day-old animals. B, genotypic characterization of wild type (NN), heterozygous (Nj), and jj animals. Following DNA extraction the region of the genome corresponding to the mutation was amplified by PCR. The amplification conditions were 94°C for 3 min, 35 cycles of 94°C for 1 min, 55°C for 1 min, 72 °C for 1 min, followed by a final step at 72°C for 5 min. The product of the amplification was then digested for 1hr at 60°C with BstN1 (NEB BioLabs). The single base deletion in the mutated allele destroys the consensus sequence of the restriction enzyme and prevent the amplicon digestion. Gel electrophoresis allows to discriminate samples from NN rats (2 bands: 231 and 80 bp), Nj rats (3 bands: 311, 231 and 80 bp) and jj rats (1 band, undigested, 311bp). C, total serum bilirubin concentration strongly increases after birth in jj animals, while NN and Nj animals have low total serum bilirubin concentration throughout development. Total bilirubin concentrations in Nj animals were very close or slightly higher to those measured in NN animals (p-value for differences between NN and Nj were 0,051 and 0,025 for 9-day-old and 17-day-old animals, respectively, one tail student t test for unequal variance). P2, P9, P17, P40: 2-, 9-, 17-, and 40-day-old animals, respectively. D, cerebellar weight measurements performed 2, 9, 17 and P40 days after birth show that jj animals develop a mild cerebellar hypoplasia. The cerebellar weights measured in jj animals were statistically different (p<0,015) from those measured in NN and Nj animals at P9, P17 and P40. In C & D, data are presented as means ± SD. Similar findings were published previously (Vianello et al., 2018).

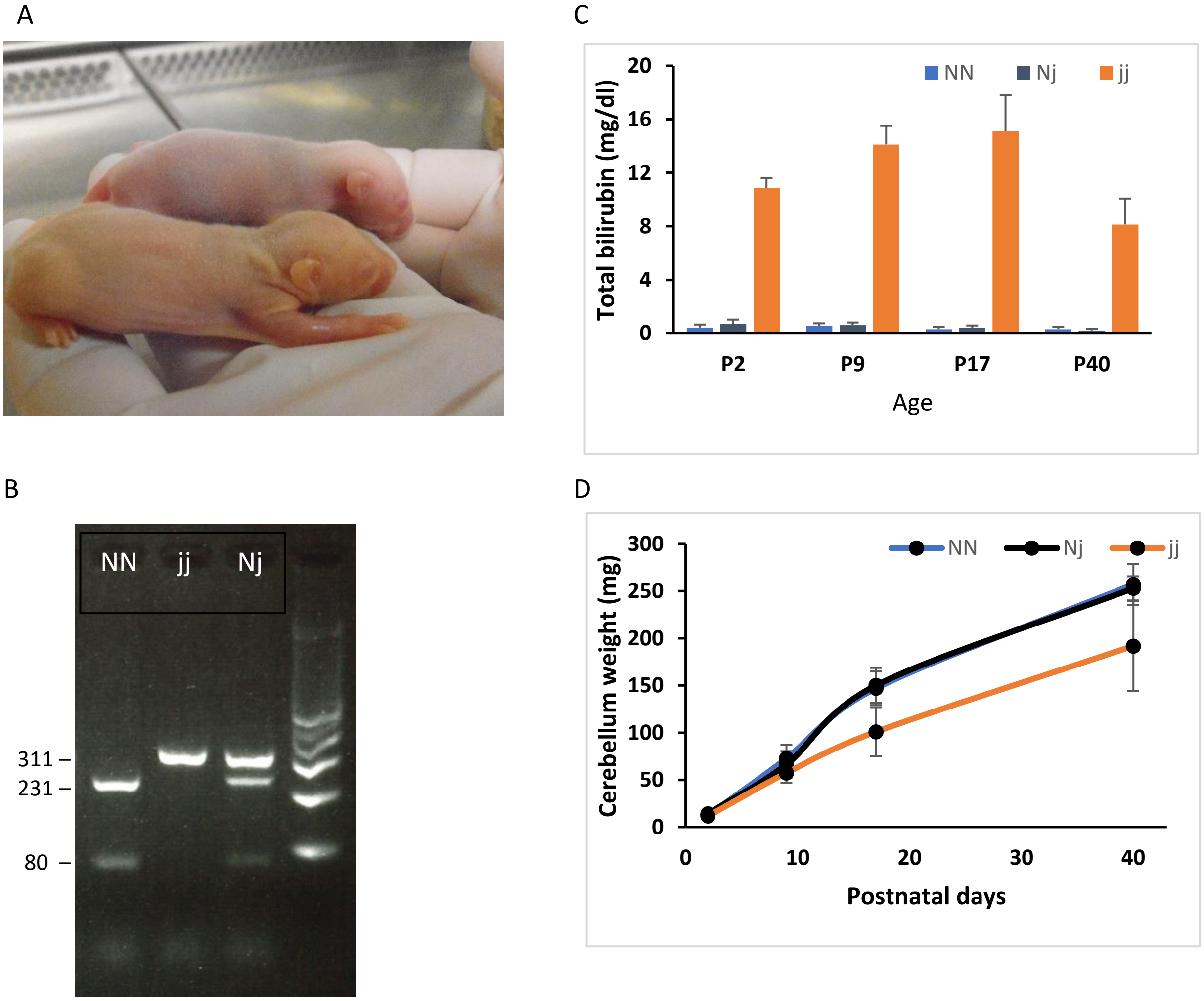

Supplementary Figure 2. Sucrose plasma concentration x time curves in eight-hour-old (P0) and 9-day-old (P9) animals, built separately from 26 and 32 hyperbilirubinemic(jj) and 42 and 47 normobilirubinemic animals, respectively. Data are expressed as 10^3 dpm.µl$^{-1}_{plasma}$ /dpm.µl$^{-1}_{injected\ solution}$. The injected solution volume-to-animal weight ratio between animals were kept constant. Data were fitted with rational model equations, using Curve Expert program.

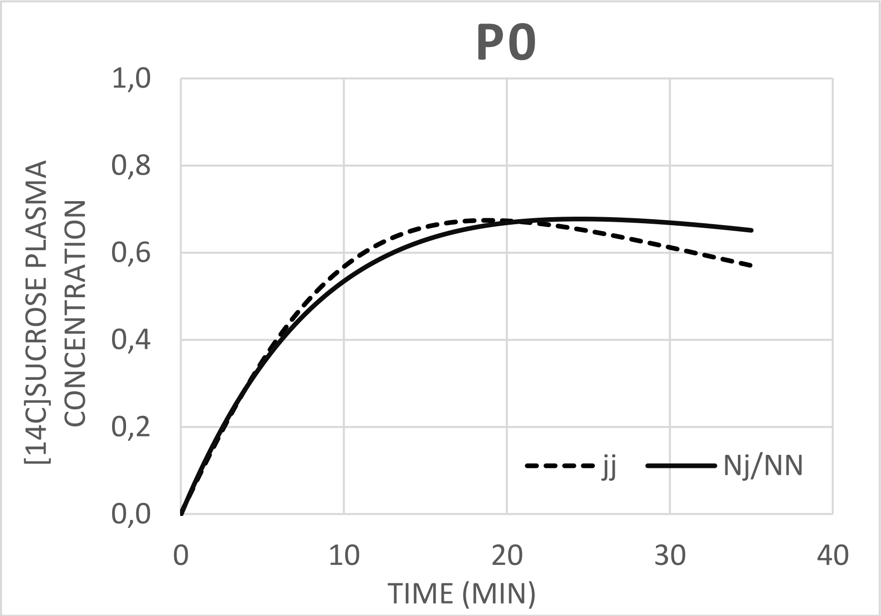

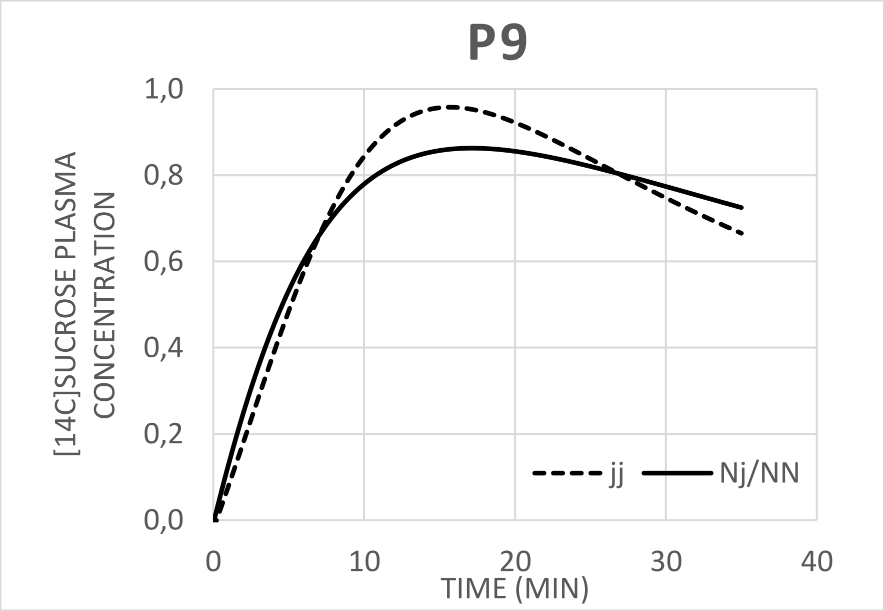

Supplementary Figure 3. Extent of the vascular network in 9-, 18-, 70-day-old (P9, P18, P70, respectively) normobilirubinemic (Nj) and hyperbilirubinemic (jj) animals. The cerebral structures and the regions of interest are depicted in Figure 1. Each field analyzed had a surface of 1,42 mm^2. Data are expressed as mean ± SD, n=6. *: $p<0.05$, statistical difference between Nj and jj structures, Anova followed by Sidak's multiple comparison test.

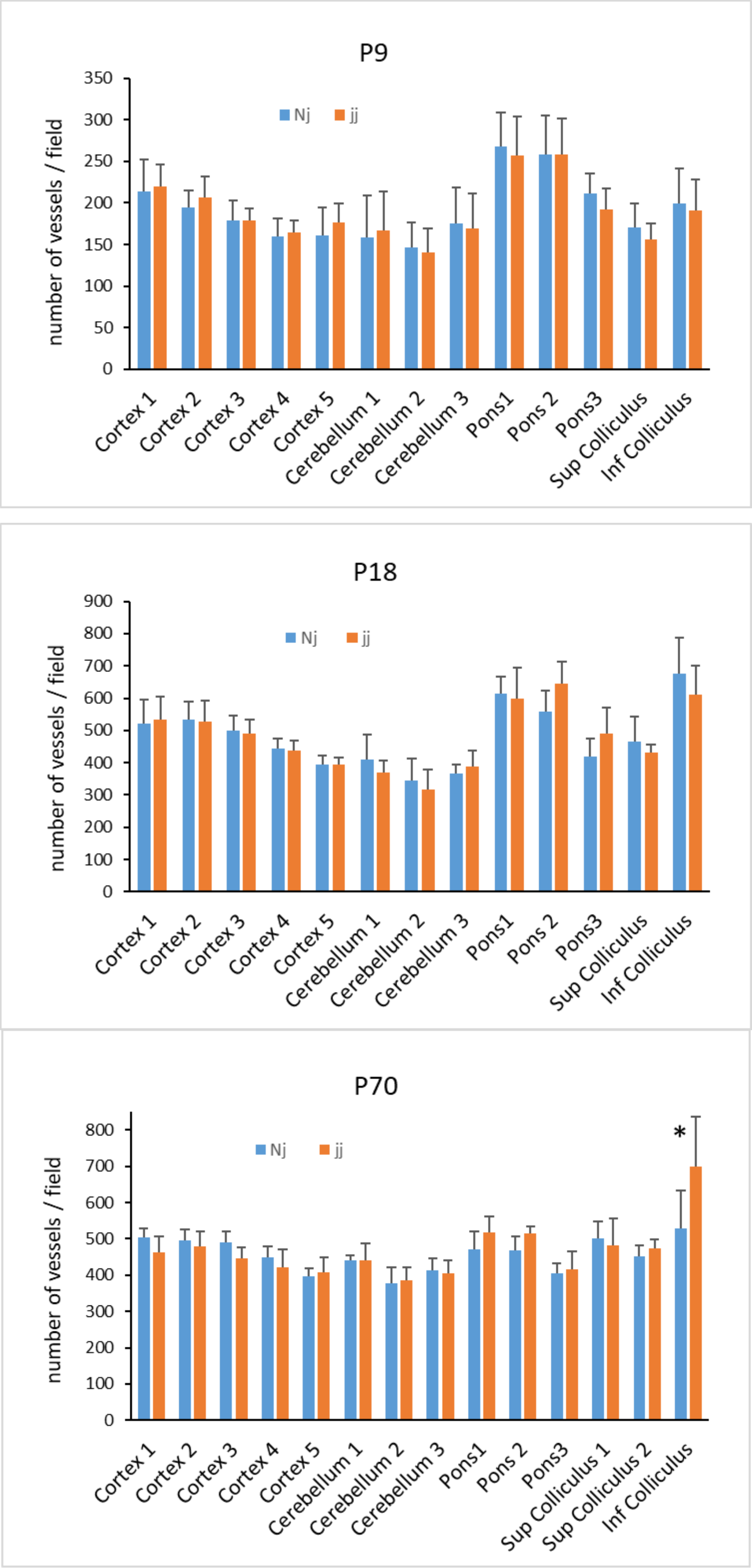

Supplement: Supplementary file 1 — Additional file 1. Additional figures S1, S2 and S3. [file 12987_2022_332_MOESM1_ESM.pdf]
