# Supplementary material for: Vascular network expansion, integrity of blood–brain interfaces, and cerebrospinal fluid cytokine concentration during postnatal development in the normal and jaundiced rat
Source: Fluids Barriers CNS. 2022 Jun 7;19:47. doi: 10.1186/s12987-022-00332-0 (PMC9172137; doi:10.1186/s12987-022-00332-0)

Supplementary figure 4. Profiles of cytokine abundance in CSF and plasma of wild type animals. Cytokines are listed in decreasing concentration order in 9-day-old (P9) animals. The same order is kept for 1-day old (P1) and 17-day-old (P17) animals. Note that analyte order differs between CSF and plasma. Data are expressed as pg/ml (mean of 4, 8 and 9 animals for 1-, 9-, 17-day-old animals, respectively). Red arrows in P1 panels highlight the specificities observed at that stage by comparison to the other two later stages.

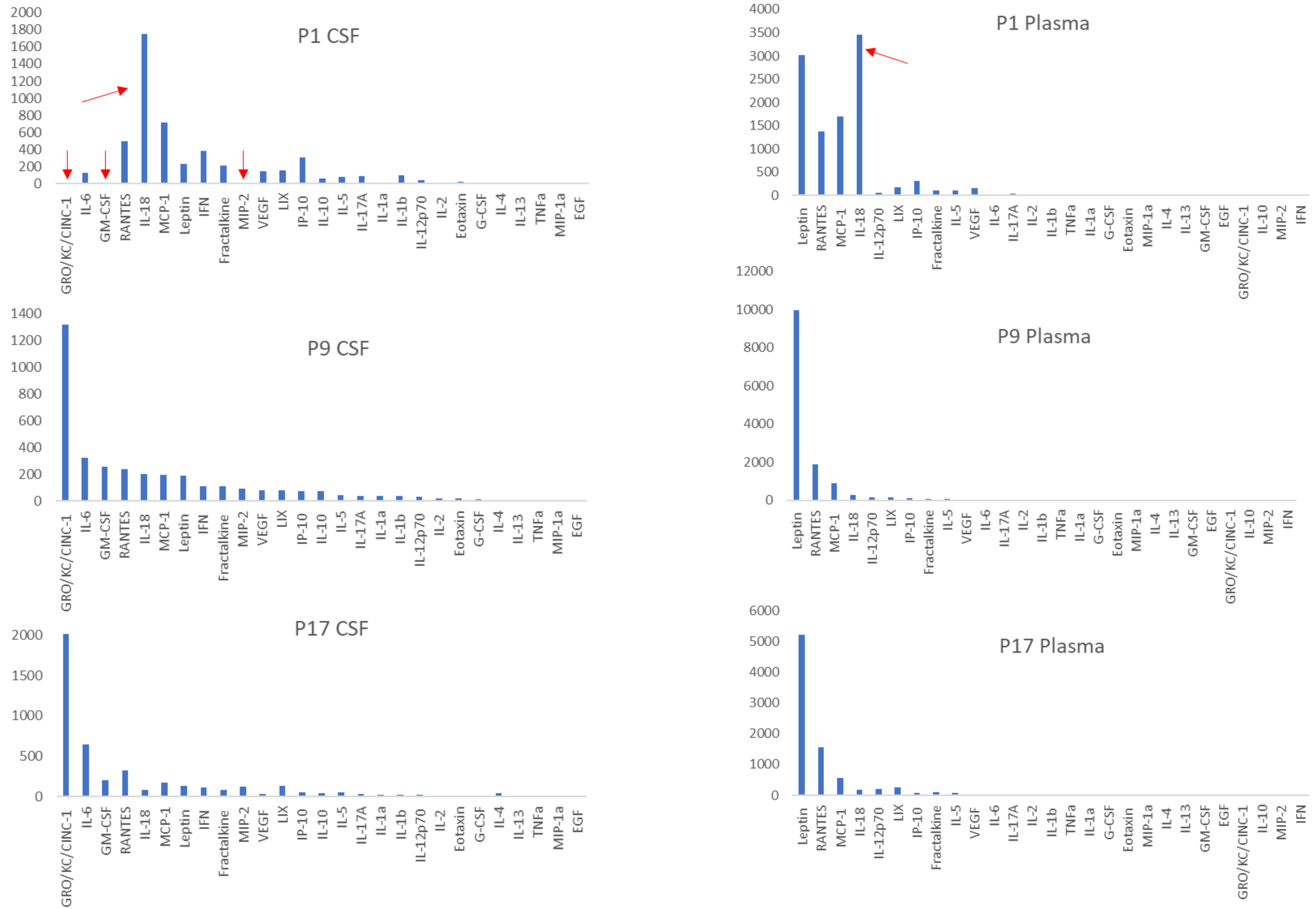

Supplementary Figure 5. Developmental profiles of cytokines in the plasma of wild-type animals. Data are expressed as pg/ml (mean ± SD of 4, 8 and 9 animals from 1-, 9- and 17-day-old animals, respectively). *, **, ***: p<0.05, p<0.01, p<0.001, respectively, Anova followed by muticomparison Tuckey test. IL-10, MIP-2, GM-CSF, IFNγ, GRO/CINC-1 were not detected at any stages. Abbreviations : P1, P9, P17: 1-,9- ,17-day-old animals, respectively

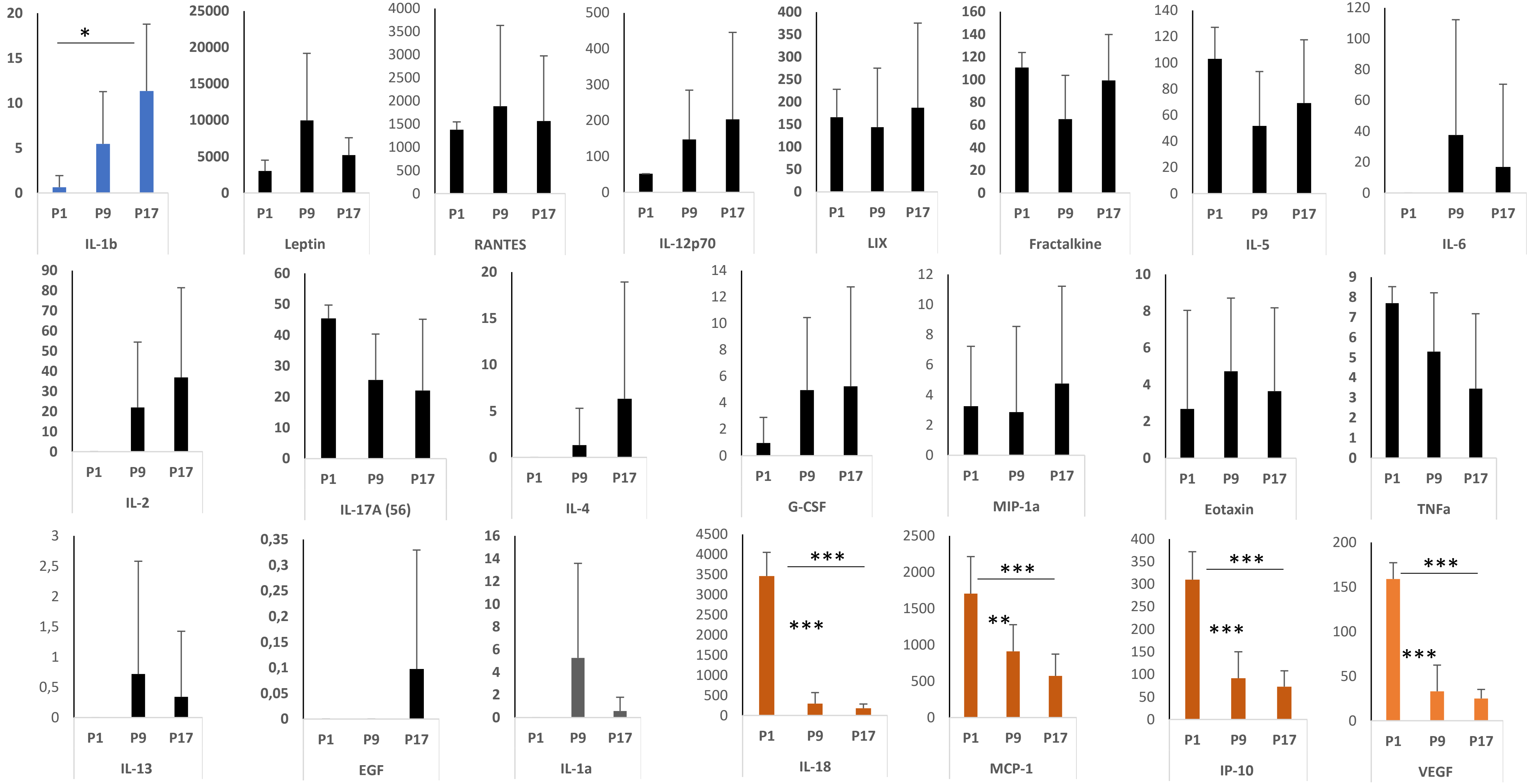

Supplementary Figure 6: Influence of hyperbilirubinemia on plasma cytokine content in seventeen-day-old animals. Only cytokines for which differences close to statistical significance were observed between animal groups are shown. Data are expressed as pg/ml, (mean ± SD from 9 animals). * p<0.05, Anova followed by Tuckey test. NN: wildtype genotype, Nj: heterozygous genotype, jj: homozygous phenotype.

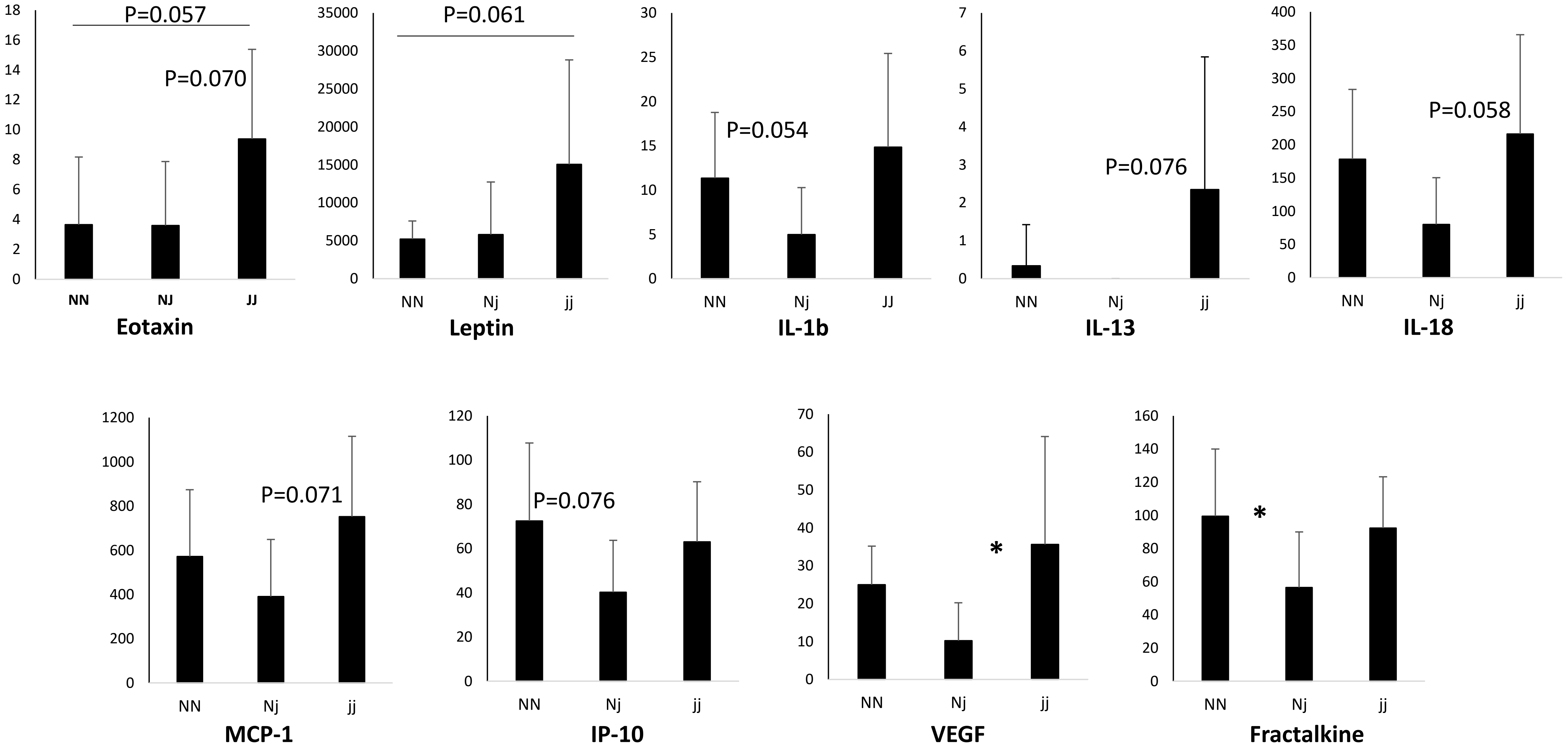

Supplement: Supplementary file 2 — Additional file 2. Additional figures S4, S5 and S6. [file 12987_2022_332_MOESM2_ESM.pdf]
